# Single-nucleus RNA sequencing reveals heterogeneity among multiple white adipose tissue depots

**DOI:** 10.1093/lifemeta/load045

**Published:** 2023-11-21

**Authors:** Limin Xie, Wanyu Hu, Haowei Zhang, Yujin Ding, Qin Zeng, Xiyan Liao, Dandan Wang, Wanqin Xie, Hannah Xiaoyan Hui, Tuo Deng

**Affiliations:** National Clinical Research Center for Metabolic Diseases, and Department of Metabolism and Endocrinology, The Second Xiangya Hospital of Central South University, Changsha, Hunan 410011, China; Key Laboratory of Diabetes Immunology, Ministry of Education, and Metabolic Syndrome Research Center, The Second Xiangya Hospital of Central South University, Changsha, Hunan 410011, China; National Clinical Research Center for Metabolic Diseases, and Department of Metabolism and Endocrinology, The Second Xiangya Hospital of Central South University, Changsha, Hunan 410011, China; Key Laboratory of Diabetes Immunology, Ministry of Education, and Metabolic Syndrome Research Center, The Second Xiangya Hospital of Central South University, Changsha, Hunan 410011, China; The First Affiliated Hospital, Department of Orthopedics, Hengyang Medical School, University of South China, Hengyang, Hunan 421001, China; National Clinical Research Center for Metabolic Diseases, and Department of Metabolism and Endocrinology, The Second Xiangya Hospital of Central South University, Changsha, Hunan 410011, China; Key Laboratory of Diabetes Immunology, Ministry of Education, and Metabolic Syndrome Research Center, The Second Xiangya Hospital of Central South University, Changsha, Hunan 410011, China; National Clinical Research Center for Metabolic Diseases, and Department of Metabolism and Endocrinology, The Second Xiangya Hospital of Central South University, Changsha, Hunan 410011, China; Key Laboratory of Diabetes Immunology, Ministry of Education, and Metabolic Syndrome Research Center, The Second Xiangya Hospital of Central South University, Changsha, Hunan 410011, China; National Clinical Research Center for Metabolic Diseases, and Department of Metabolism and Endocrinology, The Second Xiangya Hospital of Central South University, Changsha, Hunan 410011, China; Key Laboratory of Diabetes Immunology, Ministry of Education, and Metabolic Syndrome Research Center, The Second Xiangya Hospital of Central South University, Changsha, Hunan 410011, China; National Clinical Research Center for Metabolic Diseases, and Department of Metabolism and Endocrinology, The Second Xiangya Hospital of Central South University, Changsha, Hunan 410011, China; Key Laboratory of Diabetes Immunology, Ministry of Education, and Metabolic Syndrome Research Center, The Second Xiangya Hospital of Central South University, Changsha, Hunan 410011, China; NHC Key Laboratory of Birth Defect for Research and Prevention, Hunan Provincial Maternal and Child Health Care Hospital, Changsha, Hunan 410028, China; School of Biomedical Sciences, The Chinese University of Hong Kong, Hong Kong 999077, China; National Clinical Research Center for Metabolic Diseases, and Department of Metabolism and Endocrinology, The Second Xiangya Hospital of Central South University, Changsha, Hunan 410011, China; Key Laboratory of Diabetes Immunology, Ministry of Education, and Metabolic Syndrome Research Center, The Second Xiangya Hospital of Central South University, Changsha, Hunan 410011, China; Clinical Immunology Center, The Second Xiangya Hospital of Central South University, Changsha, Hunan 410011, China

**Keywords:** white adipose tissue, adipose tissue heterogeneity, adipocyte subpopulations, snRNA-seq

## Abstract

Regardless of its anatomical site, adipose tissue shares a common energy-storage role but exhibits distinctive properties. Exploring the cellular and molecular heterogeneity of white adipose tissue (WAT) is crucial for comprehending its function and properties. However, existing single-nucleus RNA sequencing (snRNA-seq) studies of adipose tissue heterogeneity have examined only one or two depots. In this study, we employed snRNA-seq to test five representative depots including inguinal, epididymal, mesenteric, perirenal, and pericardial adipose tissues in mice under physiological conditions. By analyzing the contents of main cell categories and gene profiles of various depots, we identified their distinctive physiological properties. Immune cells and fibro-adipogenic progenitor cells (FAPs) showed dramatic differences among WAT depots, while adipocytes seemed to be conserved. The heightened presence of regulatory macrophages and B cells in pericardial adipose tissues implied their potential contribution to the preservation of coronary vascular function. Moreover, the selective aggregation of pericytes within mesenteric adipose tissue was likely associated with the maintenance of intestinal barrier homeostasis. Using a combination of RNA sequencing and snRNA-seq analysis, the major subpopulations of FAPs derived from these depots determined the site characteristics of FAPs to a certain extent. Our work establishes a systematic and reliable foundation for investigating the heterogeneity of WAT depots and elucidating the unique roles these depots play in coordinating the function of adjacent organs.

## Introduction

Adipose tissue is an important regulator of whole-body metabolism and energy homeostasis [1]. However, its existence is a double-edged sword, and too much or too little will have negative effects on the body [2]. Research advancements on this dynamic endocrine organ system have been necessitated by the worldwide increase in obesity and metabolic disease [3]. Adipose tissue depots are typically classified into two distinct categories, white adipose tissue (WAT) and brown adipose tissue (BAT) [4]. WAT is widely distributed throughout the body of mammals and can be divided into subcutaneous adipose tissue (SAT), visceral adipose tissue (VAT), and bone marrow adipose tissue based on its anatomical distribution [5]. Although energy storage as a conserved function of adipose tissue is shared among WAT in different depots, they may exhibit unique biological features and functions depending on the location of distribution in the adjacent organs [6–10]. Researches on adipose tissue are becoming more site-specific. For instance, VAT was divided into six parts: perirenal, gonadal, epicardial, retroperitoneal, omental, and mesenteric adipose tissue [11].

Researchers have long been interested in the heterogeneity of adipose tissue, which is typically determined by the origin and anatomical position. The morphology and function of WAT and BAT are significantly different. BAT is an energy-dissipating and heat-producing tissue, and brown adipocytes contain an abundance of lipid droplets and mitochondria. In contrast, WAT is designed to store lipids and energy, and white adipocytes contain a large, unilocular lipid droplet [12]. WAT at various anatomical sites has distinct functions that are related to those of neighboring organs [13]. Recent studies mainly focused on identifying the differences between SAT and VAT [14–17]. Compared to SAT, VAT is more metabolically active and less insulin sensitive, and exhibits greater lipolytic activity, along with a higher degree of pro-inflammatory characteristics [14, 15]. VAT depots from distinct locations, such as epicardial, perirenal, and mesenteric adipose tissue, were usually studied separately [18–20]. Thus, a comprehensive and integrated analysis of multiple WAT depots is lacking, especially regarding various depots of VAT.

Single-cell RNA sequencing (scRNA-seq) is a meticulous technique used widely to dissect tissue heterogeneity via the transcriptional profiling of individual cells. Many studies employed scRNA-seq technology to investigate the cellular heterogeneity of adipose tissue. Since current high-throughput scRNA-seq technology does not detect adipocytes, these studies primarily focused on the heterogeneity of stromal vascular fraction (SVF). Their main goal is to identify new subpopulations of adipose stem cells [21–26] and immune cells [27, 28], as well as disease-specific cell subpopulations within adipose tissue [29, 30]. The emergence of single-nucleus RNA sequencing (snRNA-seq) technology has successfully addressed the challenges posed by the unsuitability of large-sized adipocytes for conventional scRNA-seq analysis. This advancement has facilitated the unraveling of heterogeneity within adipocytes and the understanding of interactions between adipocytes and stromal vascular cells. Regarding BAT in both humans and mice, Sun *et al*. demonstrated that the CYP2E1^+^ALDH1A1^+^ subpopulation of adipocytes plays an essential role in regulating thermogenic function [31]. snRNA-seq analysis on epididymal WAT (EWAT) from lean and obese mice revealed the impact of obesity on differentiation and subpopulation changes of adipocytes [32]. Additionally, with snRNA-seq analysis of the mouse inguinal WAT under chronic cold stress, Liu *et al*. revealed the changes occurring during the process of cold-induced adipose browning at single-cell resolusion [33]. To comprehensively characterize the heterogeneity of WAT, Emont *et al*. constructed a single-resolution atlas of human and mouse subcutaneous and visceral white fat across a range of body weight. The study highlights the crucial regulatory role of adipocytes in both local and systemic regulation of adipose tissue [34]. These scRNA-seq and snRNA-seq studies provide valuable insights into the cellular heterogeneity of WAT. However, since these studies primarily focused on one or two WAT depots, they are not fully equipped to address the heterogeneity of WAT depots across various anatomical sites.

In this study, we conducted snRNA-seq on WAT depots of five anatomical sites in mice under physiological conditions. Our data set reveals differences in cellular maps and cell interactions among multiple WAT depots. The variations in the re-clustering results of each cell type were elucidated in terms of marker genes, distribution characteristics, cell proportions, and enrichment pathways. Furthermore, flow cytometry and bulk RNA-seq analysis were used to confirm the differences in the expression profiles of immune cell subpopulations and the fibro-adipogenic progenitor cells (FAPs) at each WAT depot. Thus, our data serve as a valuable resource for gaining a better understanding of WAT biology and conducting more in-depth investigations into the role of specific WAT depots in maintaining the function of neighboring organs.

## Results

### Five WAT depots have distinct differences in the composition of six major cell types according to snRNA-seq

**Figure 1:**
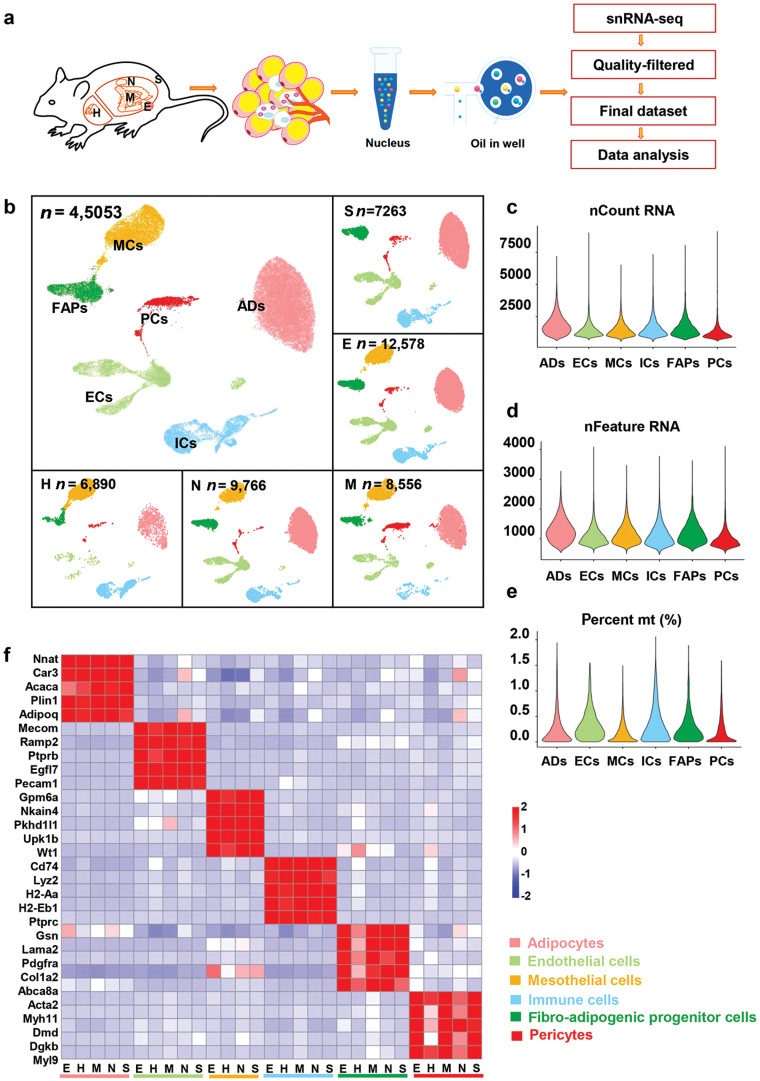
snRNA-seq displays the proportion of principal cell types in different WAT depots. (a) Workflow of our strategy to perform snRNA-seq of different WAT depots. The nuclei were derived from subcutaneous (S), epididymal (E), mesenteric (M), peri-nephritic (N), and peri-heart (H) adipose depots, which were pooled from 40 male C57BL/6J mice. Sequencing data went through the quality control process as described in the section of Metarials and methods before subsequent analysis. (b) Uniform manifold approximation and projection (UMAP) of WAT cell types and UMAP projection of all 45,053 sequenced nuclei split by depots. The embedding is based on the first 30 harmonized principal components. ADs, adipocytes; ICs, immune cells; ECs, endothelial cells; MCs, mesothelial cells; FAPs, fibro-adipogenic progenitor cells; PCs, pericytes. (c–e) Distribution of nCount RNA, nFeature RNA, and percent mt (%) in cell types. nFeature RNA, the total number of genes detected in the cell whose expression level is greater than 0; nCount RNA, the sum of the expression of all genes in the cell; percent mt (%), the percentage of mitochondrial gene expression. (f) Heatmap showing scaled average expression of selected cell-type-enriched markers in each WAT depot.

To construct the atlas of cell types in different adipose tissue depots, we sequenced the nucleus derived from EWAT, subcutaneous white adipose tissue (SWAT), mesenteric white adipose tissue (MWAT), perirenal white adipose tissue (NWAT), and peri-heart white adipose tissue (HWAT). Combined the five data sets, we obtained a total of 45,053 cells for analysis after quality control (Fig. 1a; Supplementary Fig. S1a–e). In this analysis, we performed cell annotation based on marker genes for each cluster (Supplementary Fig. S2a), resulting in the identification of six major cell types, including adipocytes, immune cells, endothelial cells, mesothelial cells, FAPs, and pericytes (Fig. 1b–e). Notably, the cell-type marker genes were very specific in each adipose tissue depot (Fig. 1f), suggesting constant cell types in different WAT depots.

**Figure 2:**
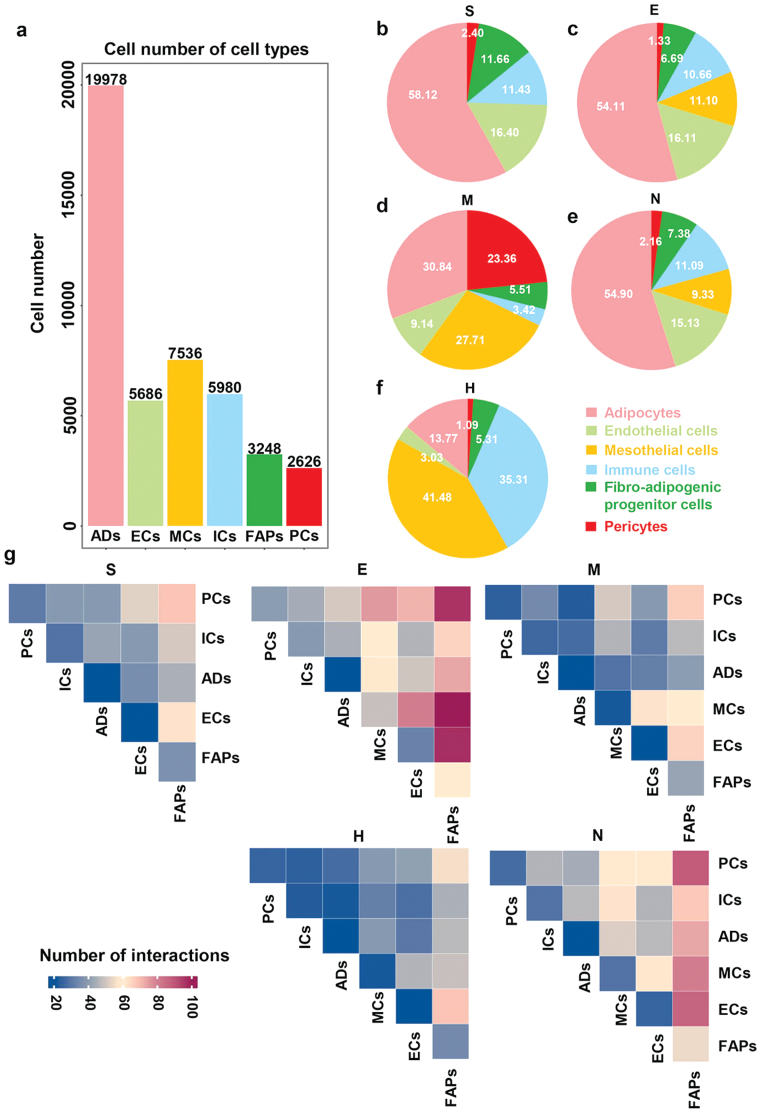
The quantity, distribution proportions, and interactions of cell types. (a) Cell number of cell types. (b–f) The fraction (relative to the total number of nuclei) of each cell type in different WAT depots. (g) CellphoneDB identified the cell–cell interactions in different WAT depots.

We compared cell type composition and distribution across depots (Figs 1b and 2a–f). In comparison to other WATs, pericytes were significantly increased in MWAT, whereas adipocytes were drastically diminished (Fig. 2d). NWAT and EWAT had comparable cell proportions (Fig. 2c and e). Immune cells represented a significant subgroup of HWAT, even outnumbering adipocytes (Fig. 2f). In addition, HWAT comprised the greatest number of mesothelial cells among the WATs identified in this study (Fig. 2f). Even though cellular fractions in different WAT depots differed dramatically, the gene expression profiles of the same cellular subpopulations remained similar.

To determine the potential interactions between cell types in five different depots in physiology states, we performed intercellular communication analysis by CellphoneDB (Fig. 2g). Overall, FAP appeared to be more active in cellular interactions compared with others, which had the richest ligand–receptor pairs, particularly with endothelial cells and pericytes. The EWAT and NWAT seem to be the depots where the majority of intercellular interactions occur, and Emont *et al*. found that the cellular interactions of EWAT were more robust than those of SWAT [34]. Together, we presented a map of WAT cells, revealing the cell compositional properties of various WATs and illustrating the relationships between cell subsets based on snRNA-seq.

### The compositions of immune cells are varying in adipose tissue at different locations

**Figure 3:**
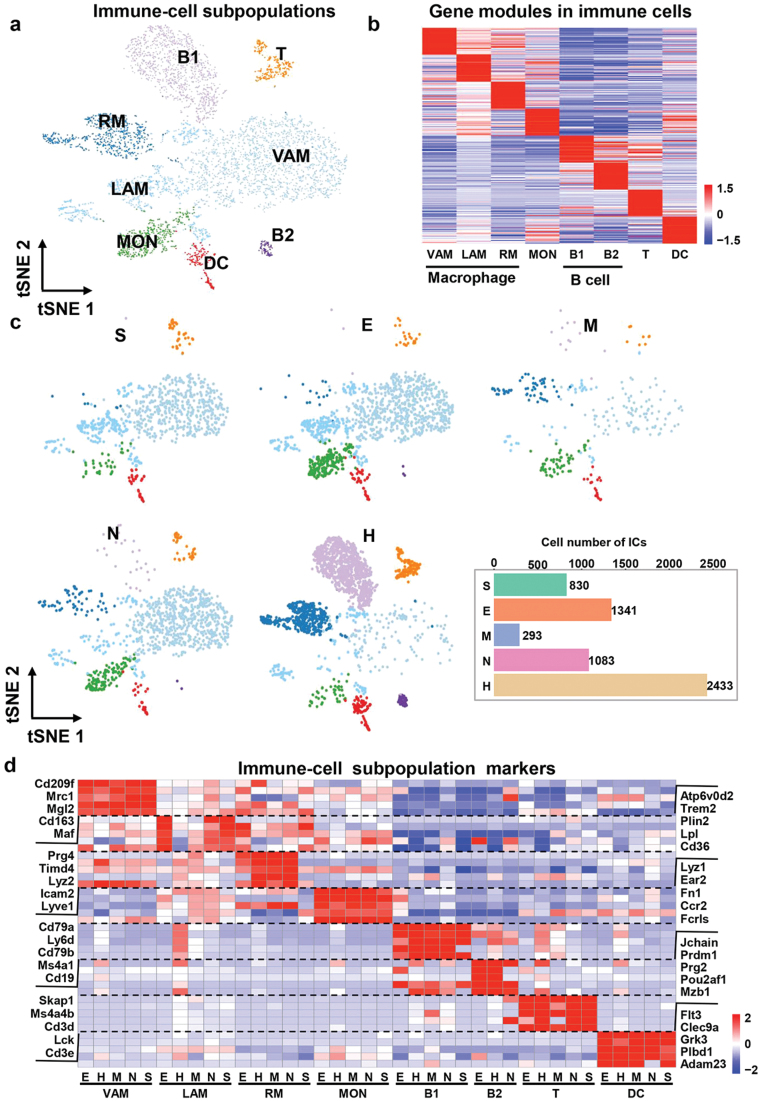
Immune cells show different compositions in different location part adipose tissues. (a) Dimensionality reduction plot of immune cells re-clustering subpopulations. The embedding is based on the first 13 harmonized principal components. VAM, perivascular macrophages; LAM, lipid-associated macrophages; RM, regulatory macrophage; MON, monocytes; B1/B2, cluster 1 or cluster 2 of B cell; T, T cell; DC, dendritic cell. (b) Heatmap showing average scaled gene module scores for the top 50 most enriched marker genes in each cluster. (c) UMAP projection of all 5980 immune cells split by depots. (d) Heatmap showing scaled average expression of immune cell subclusters marker genes in each cluster in each WAT depot.

**Figure 4:**
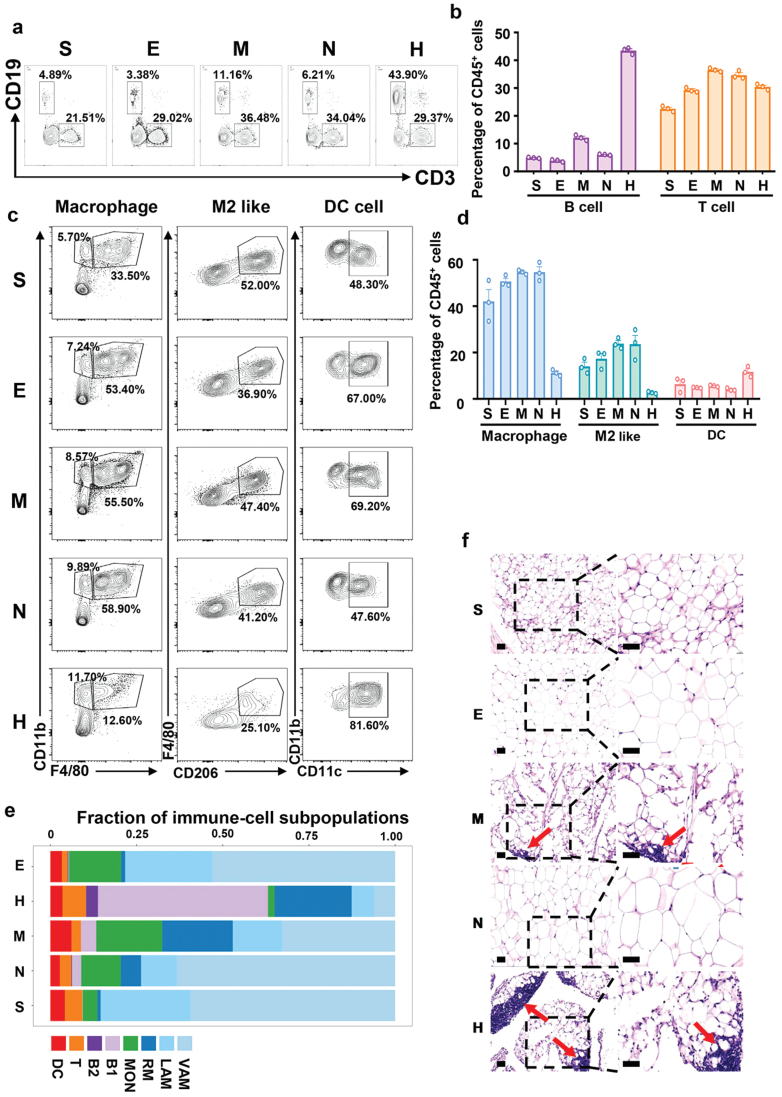
Flow cytometry experiments, subpopulation proportions of immune cells, and hematoxylin and eosin (H&E) staining of adipose tissue. (a and b) The lymphocyte proportion in different WAT depots detected by flow cytometry. Flow cytometry analysis (a) and quantification (b) of CD19^+^ (B cell) and CD3^+^ (T cell) cells in the CD45^+^ hematopoietic fraction isolated from subcutaneous, epididymal, mesenteric, peri-nephritic, and peri-heart adipose depots (*n* = 3 samples [3–7 mice/sample]/group). Data represent mean ± SEM. (c and d) The myeloid cell proportion in different WAT depots detected by flow cytometry. Flow cytometry analysis (c) and quantification (d) of CD11b^+^ and F4/80^+^ (macrophage), CD206^+^ and F4/80^+^ (M2 like macrophage), CD11b^+^ and CD11c^+^ (DC) in the CD45^+^ hematopoietic fraction isolated from subcutaneous, epididymal, mesenteric, peri-nephritic, and peri-heart adipose depots (*n* = 3 samples [3–7 mice/sample]/group). Data represent mean ± SEM. (e) The fraction of each immune cell subcluster in different WAT depots. (f) Representative images of H&E staining from subcutaneous, epididymal, mesenteric, peri-nephritic, and peri-heart adipose depots. The red arrow indicates the FALC. Scale bar: 50 μm.

We generated a precise map of 5980 immune cells, which have been arranged into eight distinct clusters. After comparing expression levels of markers (Supplementary Fig. S2b), we identified eight subpopulations into five types of immune cells including macrophages (expressing, e.g., *Adgre1*, *Lyz2*), monocytes (expressing, e.g., *Lyz1*, *Fn1*), B cells (expressing, e.g., *Cd79a*, *Cd79b*), T cells (expressing, e.g., *Cd3d*, *Cd3e*), and dendritic cells (DCs, expressing, e.g., *Flt3*, *Cd209a*) (Fig. 3a–c). The heatmap of the top 50 most specific genes for each subpopulation revealed that they were highly enriched in the subpopulation (Fig. 3b), but not all in five distinct depots (showing representative five marker genes) (Fig. 3d), indicating that the candidate marker genes are specific and conserved in different WAT locations. The different expression levels were mainly caused by subpopulation variations among the five depots. To examine the proportion of immune cells in different locations of adipose tissues, we separated the SVF fraction from five distinct fat pads and determined immune cells by flow cytometry (Fig. 4a–d). As shown in Figs 3c and 4e, HWAT contained fewer macrophages and monocytes but much more B lymphocytes, similar to the results of flow cytometry (Fig. 4a–d). Our findings and flow cytometry indicated that T cells and DCs had minimal differences among different depots.

It has been reported that the macrophage is the major immune cell in EWAT. Our research distinguished three subpopulations. Consistent with prior research on adipose tissues [32], we found a similar marker gene expression profile in our study (Fig. 3d). Subpopulation 1 highly expressed *Cd163* and *Maf*, which are markers of perivascular macrophages (VAMs). Moreover, VAM genes (e.g., *Mrc1*, *Cd209f*) were highly expressed; thus, we refer to this M2-like subpopulation as VAM [35]. Subpopulation 2 has a strong relationship with lipid-associated macrophages (LAMs), as evidenced by the expression of certain genes (*Trem2*, *Plin2*) and lipid-related genes (e.g., *Lpl*, *Cd36*) at high levels; hence, we refer to this population as LAMs [27, 32]. Subpopulation 3 exhibited a high level of *Prg4*; hence, the term regulatory macrophages (RMs) is retained [32]. RMs express another important macrophage marker *Timd4*, which is well known as tissue-resident macrophage marker [36, 37]. Silva *et al*. denoted VAM as adipose tissue-resident macrophage since VAM abundantly expressed *Timd4*. However, our study was able to clearly distinguish them [38]. Jaitin *et al*. and Sárvári *et al*. employed *Cd9* as a co-expressed marker of LAMs [27, 32], but we cannot consistently observe this occurrence. Perhaps they exhibited macrophage marker in a mixed setting of high-fat diet (HFD) and normal chow diet (ND), whereas we only examined the ND condition. We further hypothesize that *Cd9*, as an inducible gene, is only expressed in the HFD condition.

We compared the composition of macrophage subsets in various fat pads and determined that except HWAT, VAM constituted the majority of macrophage subpopulations, whereas RMs included the greatest number of macrophage subpopulations (Fig. 4e). SWAT and EWAT, in comparison to MWAT and NWAT, contained minimal RMs and a significant proportion of LAMs (Fig. 4e).

The B lymphocyte compartment contains two subpopulations, B1 and B2. Except for HWAT, each subgroup was very evenly distributed in various adipose tissues (Fig. 4a and b). As stated previously, HWAT revealed a large number of immune cells, with B lymphocytes, especially B1, being the vast majority. It may be due to peri-heart fat carrying a greater number of fat-associated lymphoid cluster (FALC) structures (Fig. 4f). B2 was essentially nonexistent in MWAT and SWAT. Except for HWAT, monocyte compartments were distributed uniformly throughout WATs. Compared with the other four adipose tissues, the ratio of monocytes in HWAT was minimal. The T lymphocyte and DC compartments were uniformly distributed among five WAT depots (Fig. 4a–d).

In conclusion, we profiled the immune cells of WATs and compared the primary immune cell subset differences between WATs. We discovered that macrophage is the largest compartment in the four WATs except for HWAT, where B lymphocytes are the predominant subtype in HWAT. VAM is the predominant macrophage subset in fat pads of the four WATs excluding HWAT, whereas RMs are the predominant macrophage subset in HWAT. Moreover, we identified a tissue-specific distribution of B lymphocyte subsets in WATs.

### Mesothelial cell populations show similar composition in different VAT depots, and endothelial cell fraction differences are limited in both VAT and SWAT under the physiological state

**Figure 5:**
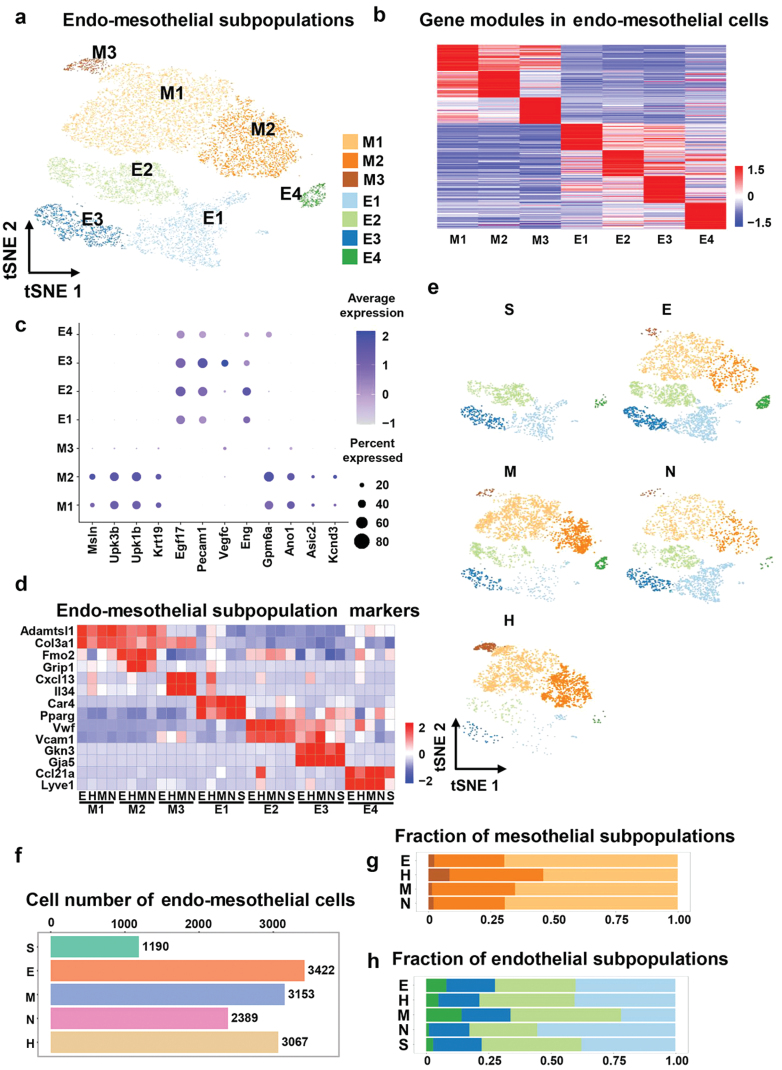
Mesothelial and endothelial cell populations show different compositions in different location part adipose tissues. (a) Dimensionality reduction plot of mesothelial and endothelial cells re-clustering subpopulations. The embedding is based on the first 15 harmonized principal components. M1–M3, mesothelial cell subpopulations; E1–E4, endothelial cell subpopulations. (b) Heatmap showing average scaled gene module scores for the top 50 most enriched marker genes in each cluster. (c) Expression of endo-mesothelial cell or their subpopulations markers across different subpopulations. Markers of mesothelial cell (*Msln*, *Upk3b*, *Upk1b*, and *Pkhd1l1*), endothelial cell (*Egfl7*, *Pecam1*, *Vegfc*, and *Eng*), and M2 subsets (*Gpm6a*, *Ano1*, *Asic2*, and *Kcnd3*) showed by feature plots. (d) Heatmap showing scaled average expression of mesothelial and endothelial cell subclusters marker genes in each cluster in each WAT depot. (e) UMAP projection of all 13,221 mesothelial and endothelial cells split by depots. (f) Cell number of mesothelial and endothelial cells in different depots. (g and h) The fraction of each mesothelial (g) and endothelial (h) cell subcluster in different WAT depots.

Mesothelial cells are specialized epithelial cells that cover the entire surface of coelom and VAT [39]. These cells synthesize various cytokines, including chemokine (C-X-C motif) ligand 1 (*CXCL1*), interleukin (*IL*)*-1*, and *IL-6*, thus enhancing the immune and inflammatory response in the cavity, and promoting the formation of FALC, a tertiary lymphoid organ structure in adipose tissue [40, 41]. Endothelial cells in adipose tissue are mostly derived from the endothelial cell portion of arteries, veins, and lymphatic vessels. According to the sequencing data, approximately 30% of the total adipose tissue cells were mesothelial and endothelial cells (Fig. 2b–f). To determine the subsets of mesothelial and endothelial cells, we performed a re-clustering analysis on all of their nuclei (*n* = 13,221) and divided them into seven distinct subpopulations (Fig. 5a). The heat map of the top 50 genes in each subset shows that there are significant gene expression clusters in each cluster (Fig. 5b).

We defined the subsets in the clusters according to the functionally characterized genes of each cluster and the relevant references. We annotated the subpopulations M1, M2, and M3 as mesothelial groups (expressing, e.g., *Msln*, *Upk3b*, *Upk1b*, and *Krt19*) and subpopulations E1, E2, E3, and E4 as endothelial cells (expressing, e.g., *Egfl7*, *Pecam1*, and *Eng*) (Fig. 5c). Specifically, as M1 highly expressed extracellular matrix genes, we denoted this subpopulation as extracellular matrix-related mesothelial cells (ERMC). As M2 highly expressed *Fmo2*, *Grip1*, and other ion regulating genes including *Gpm6a*, *Ano1*, *Asic2*, and *Kcnd3* (Fig. 5c and d), we denoted M2 as ion regulatory mesothelial cells. The M3 subpopulation was a very tiny proportion of subgroups annotated as an immune-related mesothelial cluster as they highly expressed *Cxcl13* and *Il34* genes. E1 subset highly expressed lipid homeostasis-associated genes, such as *Car4* and *Pparg*; thus, we annotated it as lipid-regulate endothelial cells. Consistent with previously reported data [32], subpopulations E2 and E3 highly expressed *Vegfc*, *Vcam1*, and *Vwf* genes, which are marker genes of the vascular endothelial cells, and the E4 subset highly expressed *Ccl21a* and *Lyve1*; thus, we defined them as lymphatic endothelial cells (LECs) (Fig. 5d).

For mesothelial cell composition analysis, four VAT depots had similar compositions, except the HWAT depot, which contained much more M3 (Fig. 5e–g). Interestingly, M3 highly expressed *Cxcl13*, a B-cell chemotaxis-related gene that plays an important role in the formation of FALCs. Previous studies have shown that pericardiac adipose tissue is a FALCs-rich adipose tissue depot [10, 41]. Comparing the endothelial cell subpopulations of the various fat pads revealed similar proportions of each category, while the MWAT depot had a greater proportion of LEC (Fig. 5e, f, and h). This result may potentially give a novel theoretical foundation for the anatomical basis of lymphatic vascular growth and malfunction in MWAT as a result of obesity [42].

### The FAP subsets show dramatic variability in different WAT depots, and the tissue characteristics are related to the enrichment of corresponding functional subsets

**Figure 6:**
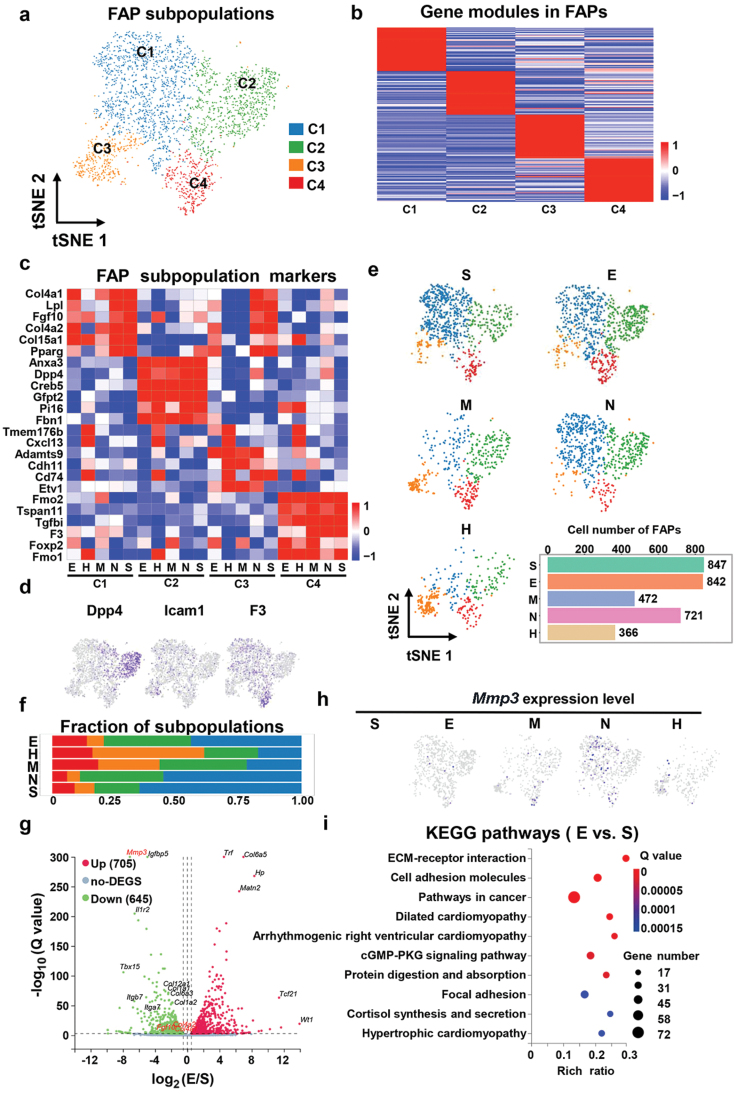
The proportion of FAP subsets in WAT depots is significantly different, and the tissue characteristics are related to the enrichment of corresponding functional subsets. (a) Dimensionality reduction plot of FAP re-clustering subpopulations. The embedding is based on the first 10 harmonized principal components. (b) Heatmap showing average scaled gene module scores for the top 50 most enriched marker genes in each cluster. (c) Heatmap showing scaled average expression of FAP subclusters marker genes in each cluster in each WAT depot. (d) Expression of FAP marker genes reported in the literature for the subsets. (e) UMAP projection of all 3248 FAP cells split by depots. (f) The fraction of each FAP subcluster in different WAT depots. (g) Volcano plot representing the differential expression genes (DEGs) between stem cells derived from epididymal adipose depot and subcutaneous adipose depot. Gray points are genes not significantly differentially expressed. Red and green points represent genes upregulated or downregulated by more than 2-fold with *P* < 0.05, respectively. (h) Expression of *Mmp3* as a depot-specific gene of FAP in different adipose tissue depots. RNA-seq differences between flow cytometry-sorted stem cells derived epididymal adipose depot and subcutaneous adipose depot. (i) KEGG pathway enrichment analysis of DEGs between stem cells derived epididymal adipose depot and subcutaneous adipose depot. The top 10 enriched terms are shown for the comparison.

FAP is a mixture of pre-adipocytes, stem cells, and fibroblasts. The re-clustering of FAP (*n* = 3248) had separated them into four distinct subsets (Fig. 6a), which we initially characterized using differential gene expression and unsupervised clustering analysis. The analysis of the top 50 most specific marker genes in each subgroup revealed significant differences in their degree of enrichment in the subgroup (Fig. 6b), indicating that the gene modules in these clusters have a distinct distribution. To study the characteristics of cell subsets after re-clustering, we screened six potential marker genes in each subpopulation and displayed the expression intensity of subgroups in different parts of adipose tissue in the form of a heat map. The results showed that cluster 1 expressed the highest levels of collagen-related marker genes, such as *Col4a1*, *Col4a2*, and *Fgf10*, and adipogenic potential marker genes, like *Lpl* and *Pparg* (Fig. 6c). According to the expression profiles of cluster 1 (C1), it had a very strong transcription similar to the “FAP2” defined by Sárvári *et al*. [32] and ICAM+ cells annotated by Merrick *et al*. [43]. Cluster 2 (C2) was marked by the expression of *Anxa3*, *Dpp4*, *Creb5*, *Gfpt2*, *Pi16*, and *Fbn1* (Fig. 6c and d); thus, we annotated them as interstitial progenitors based on previous relevant studies [43]. Cluster 3 (C3), primarily derived from the MWAT and HWAT depots, expressed the highest levels of immune-related genes, such as *Cxcl13* and *Cd74* (Fig. 6c). Interestingly, the re-clustering and experimental results of immune cells revealed that both the HWAT and MWAT depots contained the most abundant B cells (Fig. 4a, b, and e). This indicates that C3 may have played a role in the immune function of stem cells. Cluster 4 (C4) expressed the highest anti-adipogenic genes, such as *Fmo2* and *Tgfbi* (Fig. 6c); thus, we annotated this subset as adipogenesis-regulatory cells [24]. Our analysis of each cell subset proportion in different adipose tissue depots suggested that SWAT contained the largest proportion of the C1 subpopulation and a relatively lesser proportion of the C4 subpopulation (Fig. 6e and f), indicating that tissue characteristics are related to the enrichment of corresponding functional subsets. While C3 was mostly derived from HWAT (Fig. 6e and f), this manifestation may be one of the reasons why this depot contains more B cells than other depots (Fig. 4e).

The *in vitro* chemotaxis experiments revealed that adipose-derived stem cells (ASCs) derived from HWAT exhibited relatively strong chemotactic potential towards total splenocytes and T cells (Supplementary Fig. S3a). This trend is in relative concordance with the expression of chemotactic factors observed in single-cell data of FAPs from distinct WAT depots (Supplementary Fig. S3b). Additionally, ASCs from the five WAT depots exhibited great differences from *in vitro* adipogenic and myogenic differentiation experiments. Compared to other depots, ASCs from SWAT and MWAT demonstrated the strongest adipogenic and myogenic differentiation capabilities, respectively (Supplementary Fig. S3c–f).

Furthermore, snRNA-seq is based on the expression patterns of individual cells, which is valuable for cell subpopulation analysis. However, due to its limited sequencing depth, deeper sequencing is required to elucidate the differences in the FAP expression profile at each fat depot. Therefore, we used flow cytometry to sort ASCs from four different adipose tissue depots (EWAT, MWAT, SWAT, and NWAT) and then perform transcriptome sequencing. The gate logic of flow cytometry is shown in Supplementary Fig. S3e. By comparing the differentially expressed genes (log_2_FC ≥ 1 or ≤ −1, *P* ≤ 0.05) of stem cells in SWAT and EWAT, we discovered that a substantial number of fibroblast-related genes were highly expressed in SWAT stem cells (Fig. 6g). In addition, a possible marker gene for SAT progenitor cells, *Mmp3*, was discovered in both our snRNA-seq and RNA-seq data (Fig. 6g and h), indicating that our two-sequencing results are mutually validated. The Kyoto Encyclopedia of Genes and Genomes (KEGG) signaling pathway enrichment analysis of differentially expressed genes showed that more differentially expressed genes were enriched in cell matrix and adhesion signaling pathways in SWAT (Fig. 6i). Similarly, we also performed differentially expressed gene and enrichment signaling pathway analysis on the remaining paired samples (Supplementary Fig. S4a–e), and the results also showed significant heterogeneity in the gene expression profiles of stem cells in different depots of adipose tissue detected by RNA-seq. Moreover, we found that FAPs derived from SWAT were significantly different from those from other VATs in gene expression profiles (Fig. 6g; Supplementary Fig. S3d and e) and cell subpopulation components (Fig. 6f), while perirenal-FAPs in VAT were more similar to SWAT.

Overall, we identified four populations of FAPs, the proportion of FAP subsets in WAT depots was significantly different, and the tissue characteristics were correlated with the enrichment of corresponding functional subsets. In terms of organizational similarities, FAPs derived from SWAT and NWAT had more similar cell fraction composition and gene expression features than FAPs derived from the other three VATs.

### Adipocytes derived from different adipose tissue depots show relatively limited heterogeneity in the physiological state

**Figure 7:**
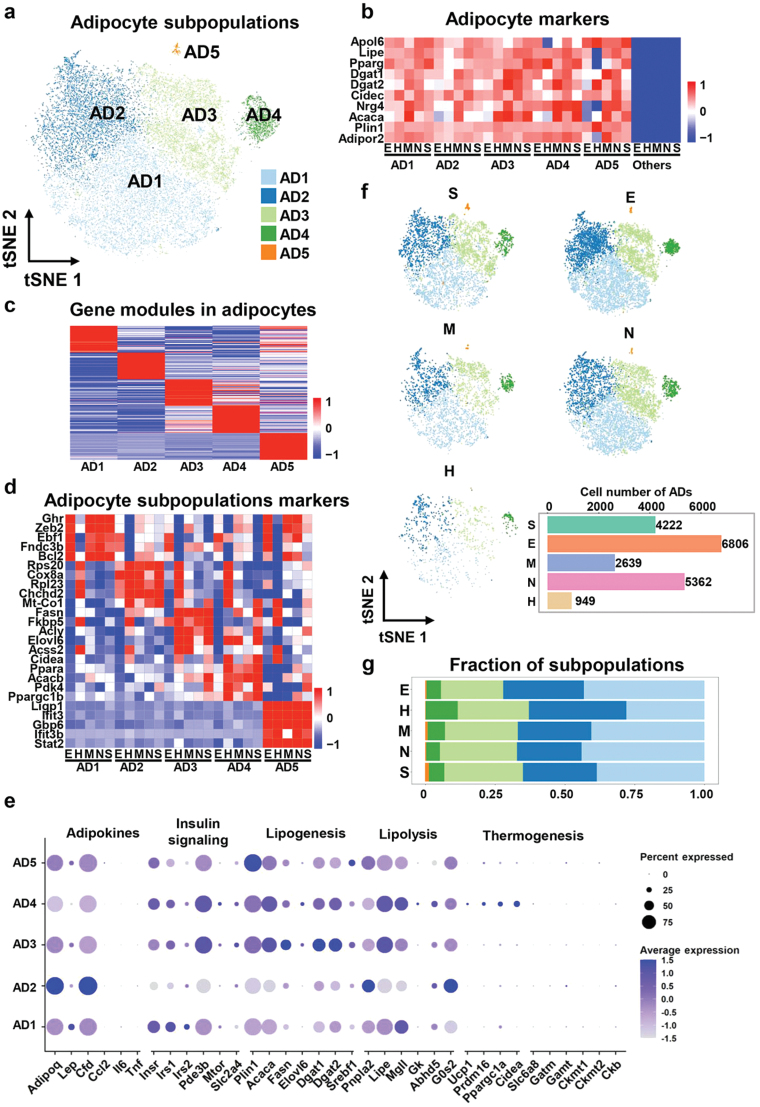
Classic adipocyte subset is the predominant adipocytes in WAT, and a subpopulation resembling brown adipocytes in peri-heart adipose. (a) Dimensionality reduction plot of adipocytes re-clustering subpopulations. The embedding is based on the first 20 harmonized principal components. (b) The expression levels of classical adipocyte marker genes in subpopulations. (c) Heatmap showing average scaled gene module scores for the top 50 most enriched marker genes in each cluster. (d) Heatmap showing scaled average expression of adipocytes subclusters marker genes in each cluster in each WAT depot. (e) Gene expression levels related to adipokine secretion, insulin signaling, lipogenesis, lipolysis, and thermogenesis in different subclusters of adipocytes. (f) UMAP projection of all 19,978 adipocytes split by depots. (g) The fraction of each adipocyte subcluster in different WAT depots.

Adipocytes made up the largest population of nuclei (*n* = 19,978, 44.34% of all detected barcodes) in WAT, and re-clustering separated the adipocytes into five distinct subsets (Fig. 7a). To study the gene expression characteristics of various subsets of adipocytes in distinct adipose tissue depots, we evaluated the expression of several typically expressed genes in adipocytes and compared them with the remaining nuclei of other cell types. We found that almost all subpopulations of adipocytes highly expressed classical adipocyte marker genes, such as *Apol6*, *Lipe*, *Pparg*, *Dgat1*, *Dgat2*, *Cidec*, *Nrg4*, *Acaca*, *Plin1*, and *Adipor2*, and the same subpopulation had limited variation in different adipose tissue depots (Fig. 7b). Our result implied that our identified adipocyte cell types were accurate and that adipocyte heterogeneity was not evident in physiological states. We further analyzed the differences in the overall gene expression of each subgroup, and found that each subgroup had higher specificity in gene expression patterns (Fig. 7c). To evaluate the biological features of the subpopulations we annotated and to further uncover possible marker genes, we conducted KEGG pathway analysis and Gene Ontology (GO) enrichment analysis using highly expressed genes of each cluster (Supplementary Fig. S5a–e). In addition, we also examined the expression levels of genes involved in major metabolic activities of adipocytes, including adipokine secretion, insulin signaling, lipogenesis, lipolysis, and thermogenesis, across different subpopulations (Fig. 7e). It is worth noting that there were certain differences in the expression of these genes among subpopulations across various adipose depots (Supplementary Fig. S6a–e). The AD1 subpopulation was annotated as a regulation metabolism cluster for highly expressed genes involved in the positive regulation of adipocyte biological behavior, such as *Fnda3b* and *Bcl2*. Furthermore, GO enrichment analysis revealed that this subpopulation’s highly expressed genes were enriched in Wnt signaling pathways (Fig. 7d; Supplementary Fig. S4a). Enrichment analysis suggested that the AD2 subpopulation was considerably enriched in the energy derivation by oxidation of organic compounds and oxidative phosphorylation signaling pathway; thus, they have been annotated as a cluster with active oxidative respiratory energy metabolism (Fig. 7d; Supplementary Fig. S4b). The AD2 subpopulation exhibited relatively high expression of adiponectin (*Adipoq*) and adipsin (*Cfd*) (Fig. 7e). The AD3 and AD4 subpopulations were annotated as lipid-handling subsets due to their similarity in fatty acid metabolic properties and gene expression profiles (Fig. 7c; Supplementary Fig. S4c and d). Additionally, the AD3 and AD4 subpopulations displayed similar expression patterns in terms of these adipocyte activities. However, AD4 showed relatively high expression of certain thermogenesis-related genes, and the KEGG pathway analysis also indicated an enrichment of thermogenesis pathways in the AD4 subpopulation (Fig. 7e; Supplementary Fig. S4d). In contrast, the AD5 subpopulation represented a negligible proportion of subgroups annotated as immune-related stress response clusters due to their high expression of *Ligp1*, *Ifit3*, *Gbp6*, and *Stat2* genes. Using tSNE to display the re-clustering results of adipocytes in different adipose tissue depots, we found that there were no depot-specific subgroups in these clusters (Fig. 7f). Furthermore, our statistics on the proportion of cell subsets in different samples suggested that the proportion of each subgroup varied only slightly among different adipose tissue depots (Fig. 7g).

Consequently, we discovered five distinct subsets of adipocytes in WAT, demonstrated that various subsets have substantially distinct gene expression profiles, and inferred their distinct functional properties based on signaling pathway enrichment analyses. Nevertheless, different depots contained comparable proportions of each subpopulation, indicating that adipocyte heterogeneity was not prominent in the physiological state.

## Discussion

In this study, we utilized optimized snRNA-seq technology to perform transcriptomic sequencing at the single-nucleus level on WATs from five anatomical sites. This approach allowed us to generate atlases for multiple WAT depots and dissect the heterogeneity in terms of cellular composition, interactions, and transcriptional features among different fat depots. The data unveiled a diverse landscape of cell types, including adipocytes, immune cells, endothelial cells, mesothelial cells, FAPs, and pericytes.

For the analysis of the cellular composition, the heterogeneity of different parts of adipose tissue is readily apparent, and we discovered that the heterogeneity of immune cells, mesothelial cells, and FAPs is particularly pronounced, whereas the heterogeneity of endothelial cells and adipocytes is relatively minor. SWAT is rich in adipogenic and extracellular matrix-forming C1 subsets of FAPs, which is consistent with the adipogenic biology characteristics of its derived stem cells [44]. Although NWAT belongs to VAT, its FAP components and gene expression profiles are similar to those of SWAT, providing new and conclusive evidence for the previous report that NWAT is intermediate between SWAT and VAT [45].

After performing a re-clustering analysis of immune cells, the immune cells were categorized into macrophages (VAM, LAM, RM), monocytes, DCs, T cells, and B cells. *Cd163* is a well-known M2 macrophage classification biomarker. Here, we classified *Cd163*^+^ macrophages as VAM (*cMaf* drive), consistent with previously reported data, and we referred to VAM as M2-like macrophages. As previously reported [35], VAM is predominantly embryonically derived because it expresses high levels of *Timd4*. However, in our study, we distinguished between VAM and tissue-resident macrophages. The co-expression of *Prg4* and *Timd4* indicates that RMs are tissue-resident macrophages in a physiological state [36]. This result confirms that mesothelial cells are a distinct fraction of VAT. In addition, pericytes exhibit a preferential enrichment in MWAT. Given the critical role of pericytes in upholding barrier integrity, these findings imply their profound significance as indispensable cellular components involved in the maintenance of intestinal barrier function. Physiologically, LAMs exhibit lipid-associated characteristics, but they do not express *Cd9* to a high degree. Thus, we do not recommend *Cd9* as a LAM marker, as it is only shown in certain conditions, such as obesity [27].

Since the 1980s, numerous studies have focused on the adipose tissue mesothelial cells, which form a monolayer on the surface of VAT but are absent from SWAT, and contribute to inflammation, fibrosis, adipocyte development, and energy metabolism [46]. Westcott *et al*. used a more specific mesothelial cell marker gene, *Krt19*, and found that *Wt1*^+^ mesothelial cells do not form adipocytes, but *Pdgfa*^+^ pre-adipocytes contribute to adipocyte development [47]. Our data confirmed that mesothelial cells are a distinct fraction of VAT, and the ERMC subpopulation exhibits classic mesothelial functions. Additionally, pericytes exhibite a preferential enrichment in MWAT. Given the critical role of pericytes in upholding barrier integrity [48], these findings imply their profound significance as indispensable cellular components involved in the maintenance of intestinal barrier function.

The intercellular communication analysis indicated that immune cells interact closely with FAPs or mesothelial cells derived from VAT in physiological homeostasis. Previous studies have shown that immune cells are important functional effector cells in adipose tissue, particularly in the development of obesity, and the main sources of inflammatory factors are cytokines secreted by pro-inflammatory macrophages and T cells from immune cells in adipose tissue [49]. Moreover, during the development of adipose tissue inflammation, there is also a significant pro-inflammatory feedback effect between adipocytes and immune cells [50]. Nevertheless, we discovered that in the physiological state, adipocyte-immune cell interactions were relatively absent and were instead carried out by mesothelial cells and FAPs. In addition, further re-clustering of mesothelial cells and FAP subpopulations revealed its immune-related subpopulations, which may be a critical determinant of the immunological characteristics of adipose tissue in its physiological state. For example, HWAT is abundant with immune-related mesothelial cells and FAP subpopulations that express abundant B-cell chemokines, and this adipose tissue is abundant in B cells. We propose that intimate interactions between mesothelial cells and FAPs with immune cells in VAT may play a significant role in the initiation of microenvironmental changes when VAT is exposed to overnutrition and other stresses.

The investigation of adipose tissue heterogeneity is of significant importance for understanding its physiological and pathological functions. Numerous studies have confirmed that adipose tissues in different anatomical locations exhibit unique functions that are often interconnected with neighboring organs. Therefore, exploring the factors contributing to adipose tissue heterogeneity is an area of compelling research interest. Currently, researches have indicated variations in the embryonic origins of adipose tissue at different anatomical sites. For example, Myf5-expressing cells within the paraxial mesoderm play a pivotal role in the development of brown and retroperitoneal adipose tissue, whereas Wt1-expressing cells in both the mesoderm and lateral mesoderm are critical for the formation of various types of adipose tissue within the abdominal and thoracic cavities [11]. Furthermore, intriguing investigations have been conducted to understand the consistency of adipose tissue concerning tissue morphology. It has been revealed that SAT derived from the same developmental source in mice exhibits significantly distinct metabolic functions in various regions due to notable differences in the distribution of nerve fibers [51]. Our previous research on mesenteric adipose tissue heterogeneity explored multiple aspects, including morphological structure, cellular composition, and gene expression, providing comprehensive insights into the underpinnings of adipose tissue heterogeneity [10]. However, despite a wealth of studies on adipose tissue heterogeneity, the fundamental question regarding the origins of adipose tissue heterogeneity remains inadequately addressed. Therefore, more extensive and profound investigations are warranted. Our present study contributes crucial foundational data towards addressing this question comprehensively.

Our study offers a novel perspective on the heterogeneity of WAT depots under physiological conditions through the application of snRNA-seq analysis across five distinct adipose tissue depots. The insights derived from this resource contribute to a deeper understanding of WAT biology, opening avenues for further investigations into the role of WAT in both health and disease. WAT holds significant implications for metabolic diseases, particularly obesity. Obesity leads to chronic inflammation in WAT, driven by factors such as adipocyte expansion and subsequent immune cell infiltration, among others [52, 53]. Recent researches [32, 34] have underscored the significant impact of obesity on the composition, interactions, and differentiation of cell types within EWAT and inguinal WAT. This highlights the remarkable plasticity and diversity of WAT depots in response to the stress induced by HFD. While this study predominantly focuses on the influence of different anatomical sites on WAT heterogeneity under physiological conditions, future research should delve deeper into elucidating the alterations induced by obesity within various WAT depots.

WAT exhibits a widespread distribution in both mice and humans. Given that mice serve as the most prevalent animal model for human disease research, they have played a pivotal role in our investigation of WAT. Nevertheless, our research interests extend beyond comprehending adipose tissue heterogeneity solely in physiological or pathological contexts in animal models. We are equally intrigued by unraveling the functional disparities within human adipose tissue. However, due to ethical constraints associated with human sample collection, it is currently not feasible to surgically obtain all five fat depots from a single individual.

## Materials and methods

### Mice

All mice used in this study were 10-week-old male C57BL/6J purchased from SLAC Laboratory Animal Company (Shanghai, China). All mice were housed in a standard specific pathogen-free environment with controlled temperature and a 12-h:12-h light/dark cycle, with free access to chow diet food and drinking water until the day of sampling. Before collecting WAT, the mice were anesthetized and infused with heparin sodium solution (77 mg/L, heparin sodium powder dissolved in 1× PBS) to remove blood from adipose tissue. Next, SWAT, NWAT, EWAT, HWAT, and MWAT were collected and the adjacent lymph nodes were removed from SWAT and MWAT. SWAT, EWAT, and NWAT from six mice were pooled for nucleus isolation and snRNA-seq experiments, MWAT from 10 mice for nucleus isolation and snRNA-seq experiments, and HWAT from 20 mice for nucleus isolation and snRNA-seq experiments.

### Nucleus isolation

The nucleus isolation protocol was based on previously described publishment [54]. After perfusion, WAT was minced to 2*2*2-mm^3^ bits and then immediately transferred into liquid nitrogen. About 0.3 g snap-frozen adipose tissues were homogenized with one stroke in 3 mL citric-acid buffer (sucrose 0.25 mol/L, citric acid 25 mmol/L) using a Dounce grinder. After 5 min of incubation on ice, add 3–4 more strokes. The homogenates were centrifuged at 500 g at 4°C for 5 min. After carefully removing the lipid layer and liquid layer, the nuclear pellet was resuspended in 1 mL citric-acid buffer and then filtered through a 70-µm and 40-µm strainer. The centrifugation process was repeated and the nuclear pellet was washed again. The nuclei were then resuspended in 70 µL citric-acid buffer. Nuclei were counted on the AOPI cell counter, and trypan blue staining was performed to identify nucleus intact.

### snRNA-seq

After nucleus separation, at least 8000 nuclei were immediately loaded onto the 10X Genomics Chromium controller (10X Genomics, PN110203). The libraries were prepared according to the manufacturer’s instructions, using 10X Genomics single cell 3ʹ Library and Gel Bead Kit V3 (10X Genomics, 1000075) and Chromium Single Cell B Chip Kit (10X Genomics, 1000074). The libraries were finally sequenced using the Illumina Novaseq 6000 sequencer at a depth of at least 20,000 reads per nucleus with a pair-end 150 bp (PE150) reading strategy (performed by CapitalBio Technology, Beijing).

### Analysis of snRNA-seq

Alignment, filtering, barcode counting, and unique molecular identifier (UMI) counting were performed with the CellRanger (10X Genomics) count module to generate a feature-barcode matrix and determine clusters. SoupX [55] software was employed for processing single-cell nuclear RNA sequencing data to remove environmental RNA, thereby enhancing data quality. A step-by-step quality-filtering process was used to remove low-quality nuclei. First, genes expressed in fewer than 10 nuclei were removed. Second, low-quality nuclei were removed by filtering on the proportion of mitochondrial reads (threshold: > 15%), the number of UMIs (threshold: < 1000), the number of detected genes (threshold: < 500), and the ratio between the number of UMIs and genes (threshold: > 2.5). Third, the nuclei were filtered by outlier detection using principal component analysis generated from quality metrics calculated in *scater* [56]. Fourth, for each sample, DoubletFinder [57] was employed to calculate doublet scores, and clusters with high doublet scores, low feature counts, or a lower number of UMIs were subsequently removed.

After quality control, Seurat [58] was employed to normalize and scale snRNA-seq data, and the top 2000 most highly variable genes were selected for clustering. In order to mitigate batch effects, data from different depots were integrated by harmony [59]. According to the harmonized pericytes and uniform manifold approximation and projection (UMAP) embedding, clusters were identified with the Louvain algorithm, 30 principal components, and a resolution of 0.2 in Seurat [58]. The marker genes were used to identify clusters as adipocytes, FAPs, mesothelial cells, endothelial cells, immune cells, or pericytes. The subsets were treated individually as a single object and re-integrated using the above method. Before re-integration, a small number of mesothelial cells in the S sample were removed because a small number of cells introduced too much variability in the integration. These subsets were re-clustered with 2000 variable genes, principal components (10–20), and resolution (0.1–0.3). The marker genes were obtained using the nonparametric Wilcoxon rank sum test with the adjusted *P* value using Bonferroni correction. Then the clustering was evaluated according to the significance of marker genes to determine the final re-clustering conditions.

CellphoneDB (version 2.0.0) [60] was used for the analysis of intercellular interaction. The snRNA-seq count data were divided into six different parts of adipose tissue files: SWAT, NWAT, EWAT, HWAT, and MWAT. CellphoneDB was used to perform statistical analysis on each file to evaluate the interaction under each condition. As mentioned above, before running CellphoneDB and performing statistical analysis on each file, the mouse gene names were converted to the human gene names.

The clusterProfiler [59] was used to analyze the enrichment pathway of adipocyte markers. The marker genes of the adipocyte cluster calculated by Seurat [58] in R were enriched in the biological pathway of GO or KEGG pathway after screening by Benjamin Hochberg’s corrected *P* value < 0.05.

### Cell culture

ASCs isolated from WATs of mice were cultured in DMEM/F12 plus 10% FBS, 1% Pen/Strap. Passage 3 cells were used for the experiments of this study. For *in vitro* adipogenesis, ASCs were used after confluency and treated with white adipocyte differentiation induction cocktail: 0.5 mmol/L 3-isobutyl-1-methylxanthine (IBMX, 15879-1G, Sigma), 1 μmol/L dexamethasone (D4902, Sigma), 170 nmol/L insulin (12585014, Gibco), followed by maintenance treatment (10 μg/mL insulin) every 48 h. The culture was carried out until Day 6–8 after induction, and then cells were collected for Oil-Red-O staining or RNA extraction. For *in vitro* myogenesis, the medium was replaced by DMEM/F12 plus 2% horse serum, 1% Pen/Strap when the cells reached 50% confluence. The culture was carried out until Day 10 after induction, and then cells were collected for Desmin staining or RNA extraction.

### Transwell experiment

*In vitro*, the chemotactic ability of different ASCs toward lymphocytes was assessed using a stem cell-conditioned medium on splenocytes. For the preparation of the conditioned medium, ASCs were incubated in DMEM/F12 for 24 h, and the conditioned medium was then harvested. 0.5 mL of conditioned medium from ASCs was placed in the lower layer, while 1 × 10^6^ splenocytes per well of the 24-well culture plate were cultured in 0.1 mL of 1640 medium loaded on the surface of the upper layer of a 5-μm Transwell insert (3421, Corning). Two hours after incubation, the upper layer and Transwell insert were carefully removed. Migrated splenocyte cells were counted.

### Immunofluorescence staining

ASCs after myogenic induction were then fixed in 4% paraformaldehyde for 15 min at room temperature. After washing in PBS, cells were incubated with 0.1% Triton-X 100 in PBS for 5 min and then blocked in PBS containing 5% bovine serum albumin (BSA) for 30 min at room temperature. After 3 washes, these cells were incubated with rabbit anti-Desmin (1:200, D162991, Sangon Biotec) antibody overnight at 4°C, followed by incubation with the fluorescence-conjugated second antibody for 60 min at room temperature. Goat anti-rabbit Alexa Fluor 488 antibody (1:200, A11034, Invitrogen) was used as a second antibody for Desmin. After washing, these cells were stained with 4ʹ,6-diamidino-2-phenylindole (DAPI) before image capture using an immunofluorescence microscope.

### Reverse transcription quantitative polymerase chain reaction

Total RNA was isolated from cells and tissues using Trizol (AG, AG21101) according to the manufacturer’s instructions. The RNA was reverse transcribed using the qPCR RT kit (AG, AG11728). Reverse transcription quantitative polymerase chain reactions (RT-qPCRs) were run using SYBR Green master mix (YESEN). Target gene expression levels were normalized to *ACTIN* or *36B4* expression. Primer sequences are listed in Supplementary Table S1.

### Flow cytometry analysis of immune cells and sorting of ASCs

WATs were obtained, and then cut into small pieces (~1 mm) and digested with 1 mg/mL Type II collagenase (Sigma-Aldrich, MO) and 1% BSA (Gibco, CA) at 37°C for 45 min with constant rocking. The cell mixture was filtered through a 70-μm filter into a single-cell suspension, followed by centrifugation at 500 g for 5 min, and stromal vascular cells were treated with a red cell lysis buffer. Appropriate cell suspensions were then hybridized with fluorescent dye-conjugated antibodies against the following mouse antigens.

For immune cells: cells were incubated with Zombie dye (BioLegend, Cat. No. 423101 or 423102) for 7 min at room temperature, followed by another 7 min with CD16/32 antibody (BioLegend, Cat. No. 101302, RRID: AB_312801), and stained with detection antibody for 7 min at room temperature. The stained cells were washed twice in PBS before analysis. Antibody-stained samples were analyzed using CYTEK Northern Lights NL-3000 or NovoCyte Quanteon. The detection antibodies include CD45 (BioLegend, Cat. No. 103132), CD3 (BioLegend, Cat. No. 100204), CD19 (BioLegend, Cat. No. 152410), CD45 (BioLegend, Cat. No. 103116), CD11b (BioLegend, Cat. No. 101228), CD11c (BioLegend, Cat. No. 117308), F4/80 (BioLegend, Cat. No. 123146), and CD206 (BioLegend, Cat. No. 141708). The acquired data were analyzed with FlowJo 10.0 software (FlowJo, RRID: SCR_008520). Cells were gated for singlets (FSC-H vs. FSC-A) and live leukocytes (Zombie-CD45^+^) at first, and then gated for CD19^+^ B cells and CD3^+^ T cells, or F4/80^+^ and CD11b^+^ macrophage, F4/80^+^, CD11b^+^, and CD206^+^ M2 like macrophage, F4/80^−^, CD11b^+^, and CD11c^+^ DC cell, and set the gates using appropriate compensation.

For ASCs: cells were incubated with Via520 (BD, FITC) for 15 min at room temperature, and then incubated with CD16/32 antibody (BioLegend Cat#101302) for another 15 min and stained with detection antibodies at 4°C for 25 min. Then the stained cells were washed twice in PBS. Antibody-stained samples were analyzed using R&D S3e. The detection antibodies include CD45 (Biolegend, FITC), TER119 (Biolegend, FITC), CD31 (Biolegend, FITC), CD140A (Biolegend, PE-CF594), and SCA-1 (BioLegend, PE-CY7). Cells were gated for singlets (FSC-H vs. FSC-A) and live Lin^−^ (Via520^−^CD45^−^CD31^−^TER119^−^) firstly, and then gated for CD140A^+^ and SCA1^+^ ASCs using appropriate compensation.

### Bulk RNA-seq analysis

Bowtie 2 [61] was performed to compare clean reads to the reference gene sequence, and the gene expression level of each sample was calculated using RSEM [62]. Reads from mouse data were aligned against the GRCm38 genome assemblies. The differential analysis of gene expression levels between different samples was conducted by DESeq2 [63]. Analysis of enriched pathways was performed using clusterProfiler [64].

### Statistical analysis

Figure legends provide statistical information when necessary. Unless otherwise specified, differences were tested with Student’s *t*-test. False discoveries in cases were adjusted for in all *P* values.

## Supplementary Material

load045_suppl_Supplementary_Figures_S1-S6_Tables_S1

## Data Availability

All the data supporting the findings described in this manuscript are available in the article and the Supplementary Materials. snRNA-seq data and bulk RNA-seq data are stored in the Gene Expression Omnibus database, with numbers GSE240186 and GSE239885, respectively.
