# Supplementary material for: Single-nucleus RNA sequencing reveals heterogeneity among multiple white adipose tissue depots
Source: Life Metab. 2023 Nov 21;2(6):load045. doi: 10.1093/lifemeta/load045 (PMC11748973; doi:10.1093/lifemeta/load045)
Supplement: load045_suppl_Supplementary_Figures_S1-S6_Tables_S1 [file load045_suppl_Supplementary_Figures_S1-S6_Tables_S1.docx]

**Supplementary information**

**Single-nucleus RNA sequencing reveals heterogeneity among multiple white adipose tissue depots**

**Limin Xie^1,2,‡^, Wanyu Hu^1,2,‡^, Haowei Zhang^4,‡^, Yujin Ding^1,2^, Qin Zeng^1,2^, Xiyan Liao^1,2^, Dandan Wang^1,2^, Wanqin Xie^5^, Xiaoyan Hui^6^, Tuo Deng^1,2,3,^***

^1^National Clinical Research Center for Metabolic Diseases, and Department of Metabolism and Endocrinology, The Second Xiangya Hospital of Central South University, Changsha, Hunan 410011, China

^2^ Key Laboratory of Diabetes Immunology, Ministry of Education, and Metabolic Syndrome Research Center, The Second Xiangya Hospital of Central South University, Changsha, Hunan 410011, China

^3^Clinical Immunology Center, The Second Xiangya Hospital of Central South University, Changsha, Hunan 410011, China

^4^The First Affiliated Hospital, Department of Orthopedics, Hengyang Medical School, University of South China, Hengyang, Hunan 421001, China

^5^NHC Key Laboratory of Birth Defect for Research and Prevention, Hunan Provincial Maternal and Child Health Care Hospital, 53 Xiangchun Road, Changsha, Hunan 410028, China

^6^School of Biomedical Sciences, The Chinese University of Hong Kong, Hong Kong 999077, China

**^‡^**These authors contributed equally to this work.

**^*^**Corresponding author. National Clinical Research Center for Metabolic Diseases, Department of Metabolism and Endocrinology, The Second Xiangya Hospital of Central South University, Changsha, Hunan 410011, China. E-mail: [dengtuo@csu.edu.cn](mailto:dengtuo@csu.edu.cn)


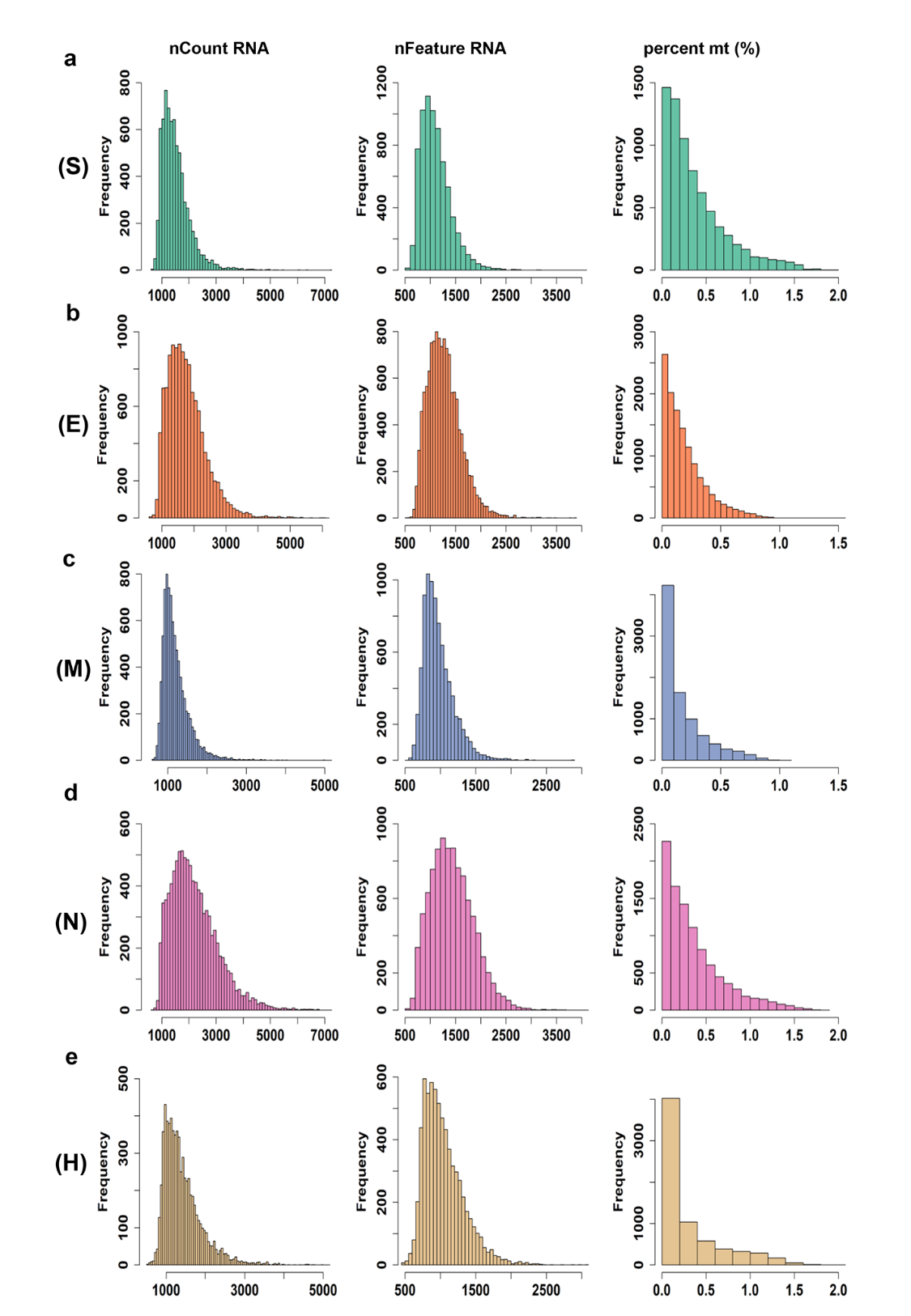


**Supplementary Figure S1** Histograms depicting the distribution of nCount RNA, nFeature RNA, and percent mt (%) in different depots. (a) subcutaneous (S), (b) epididymal (E), (c) mesenteric (M), (d) peri-nephritic (N), and (e) peri-heart (H) adipose depots. nFeature RNA, the total number of genes detected in the cell whose expression level is greater than 0; nCount RNA, the sum of the expression of all genes in the cell; percent mt (%), the percentage of mitochondrial gene expression.


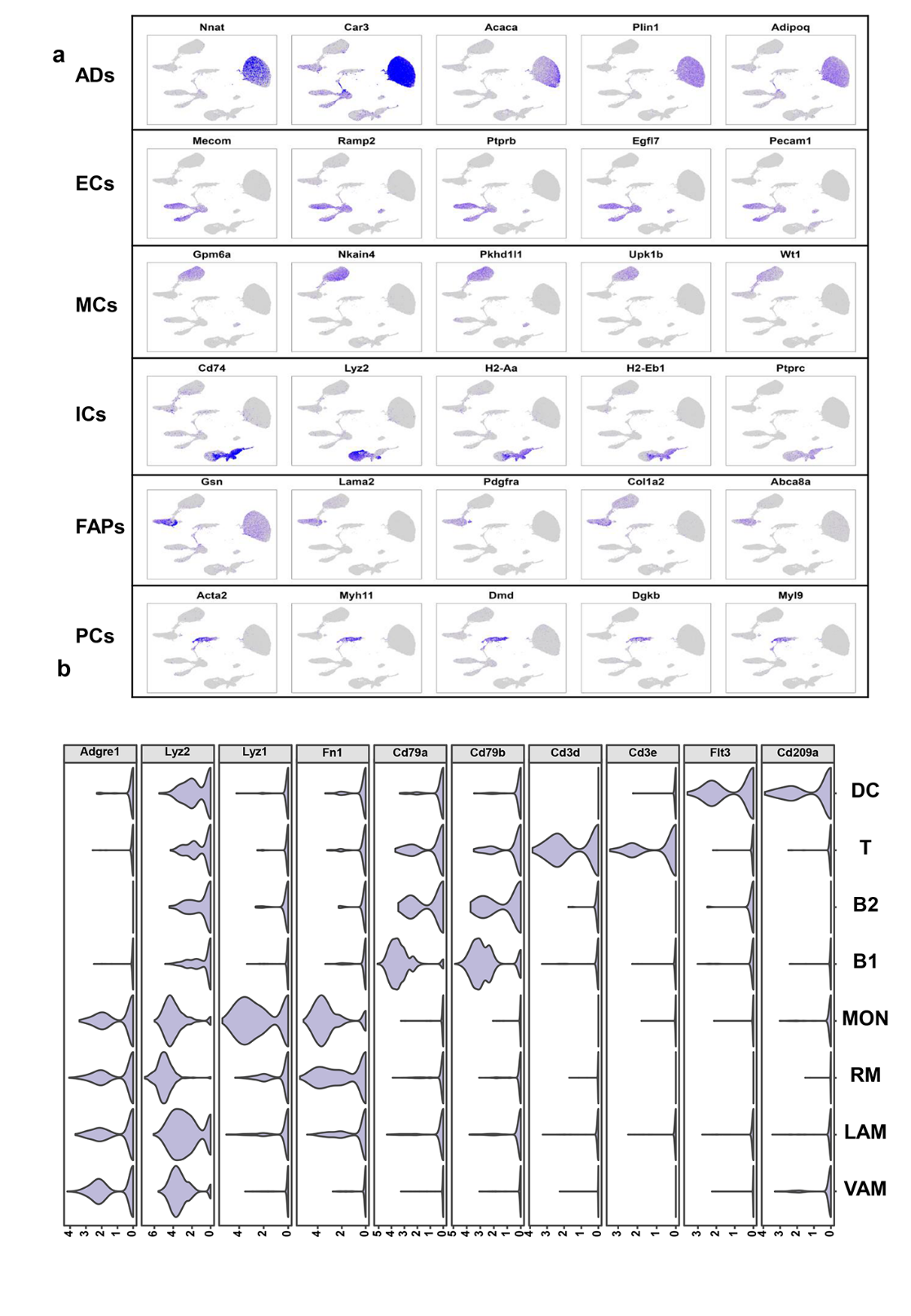


**Supplementary Figure S2** Expression profiles of marker genes for major cell types and immune cell subpopulations. (a) The plots illustrating the expression of marker genes for cell types. ADs, adipocytes; ICs, immune cells; ECs, endothelial cells; MCs, mesothelial cells; FAPs, fibro-adipogenic progenitor cells; PCs, pericytes. (b) The violin plot depicting the expression of marker genes for immune cell subpopulations. VAM, perivascular macrophages; LAM, lipid-associated macrophages; RM, regulatory macrophage; MON, monocytes; B1/B2, cluster1 or cluster2 of B cell; T, T cell; DC, dendritic cell.


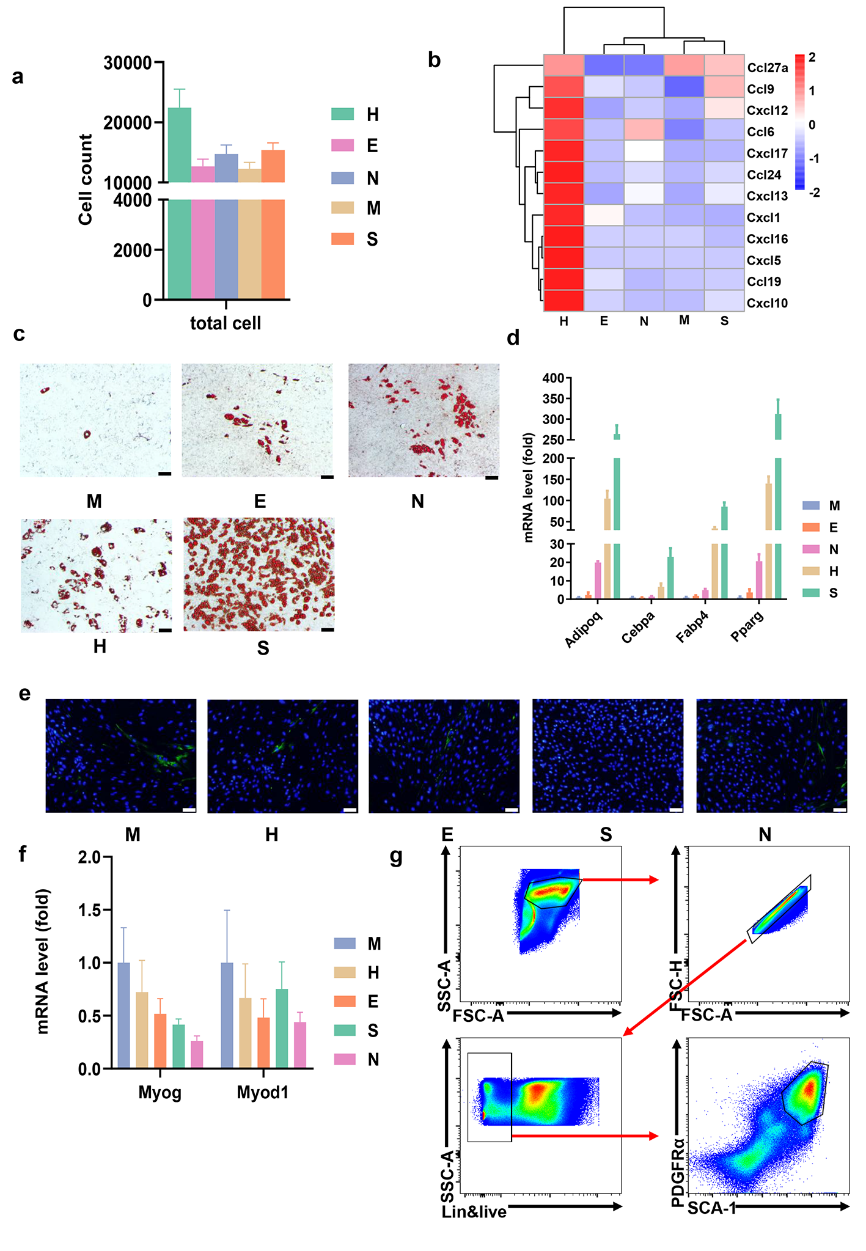


**Supplementary Figure S3** The *in vitro* experiments and flow cytometry gating strategies for ASCs. (a) Chemotaxis experiments of ASCs for lymphocytes. (b) Dot plot for the expression of chemotactic factors in FAP subgroups from five WAT depots. *n* = 3. (c) Oil-Red-O staining for ASC adipogenesis experiments. Scale bar: 200 μm. (d) The mRNA expression levels of adipogenesis-related genes. *n* = 3. (e) Immunofluorescence staining for ASC myogenesis experiments. Scale bar: 100 μm. (f) The mRNA expression levels of myogenesis-related genes. *n* = 3. (g) Gating logic of Flow cytometric sorting of adipose tissue stem cells. First, forward and side scatter density plots for identifying the cell population of interest and excluding debris. Second, forward scatter height versus forward scatter area density plot for doublet exclusion. Third, lin (*CD45*, *TER119*, *CD31*) and live parameter density excluding immune cells, hematopoietic lineages, endothelial cells, and dead cells. Fourth, the PDGFRα^+^ and SCA-1^+^ indicate the target ASC population. S, subcutaneous; E, epididymal; M, mesenteric; N, peri-nephritic; H, peri-heart adipose depots.


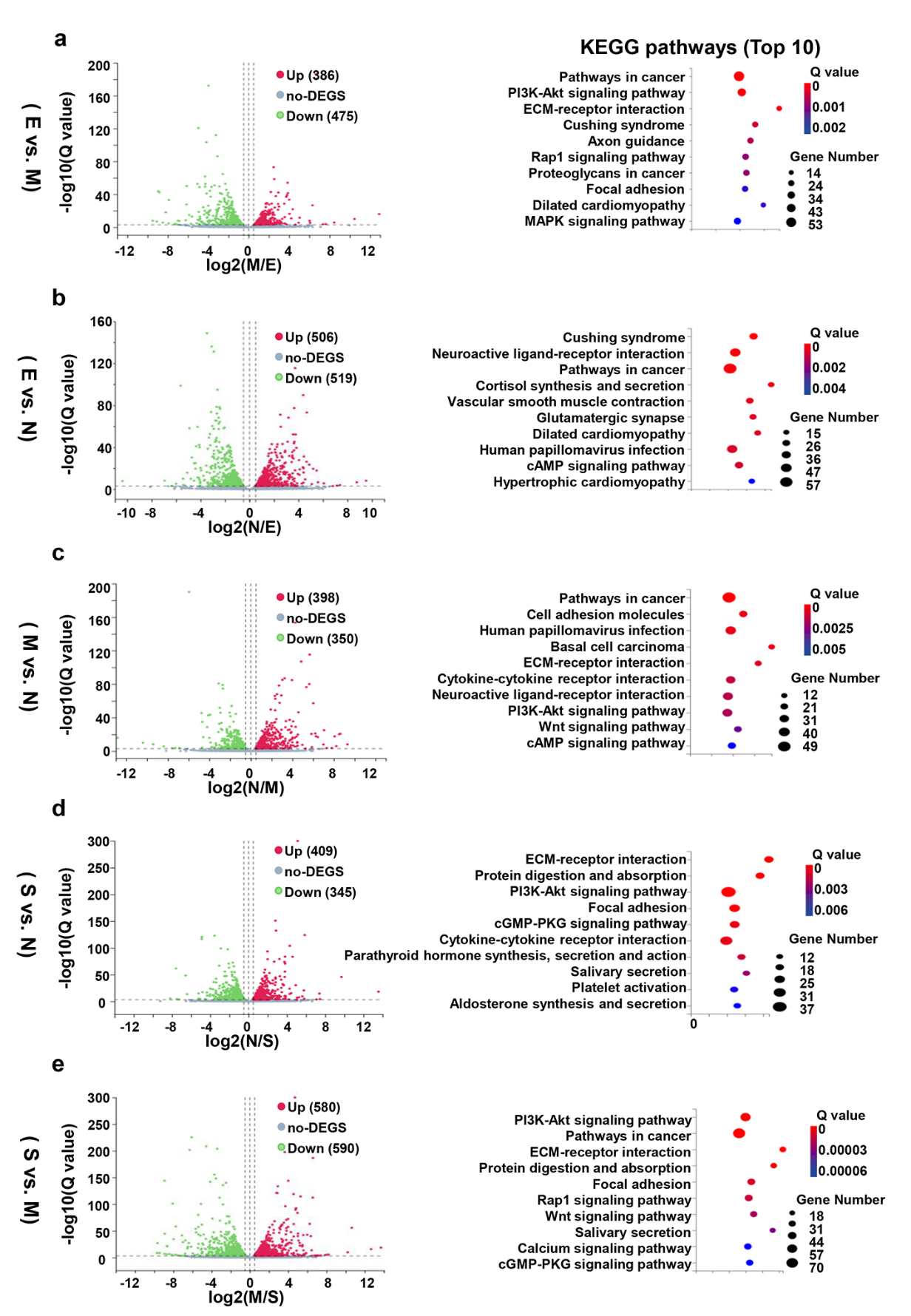


**Supplementary Figure S4** RNA-seq differences between flow cytometry-sorted ASC-derived subcutaneous (S), epididymal (E), mesenteric (M), and peri-nephritic (N) adipose depots. Volcano plot representing the differential expression genes (DEGs) and top 10 enriched KEGG pathways of DEGs between M/E (a), N/E (b), N/M (c), N/S (d), and M/S (e) respectively. Grey points are genes not significantly differentially expressed. Red and green points represent genes upregulated or downregulated by more than twofold with *P* < 0.05, respectively.


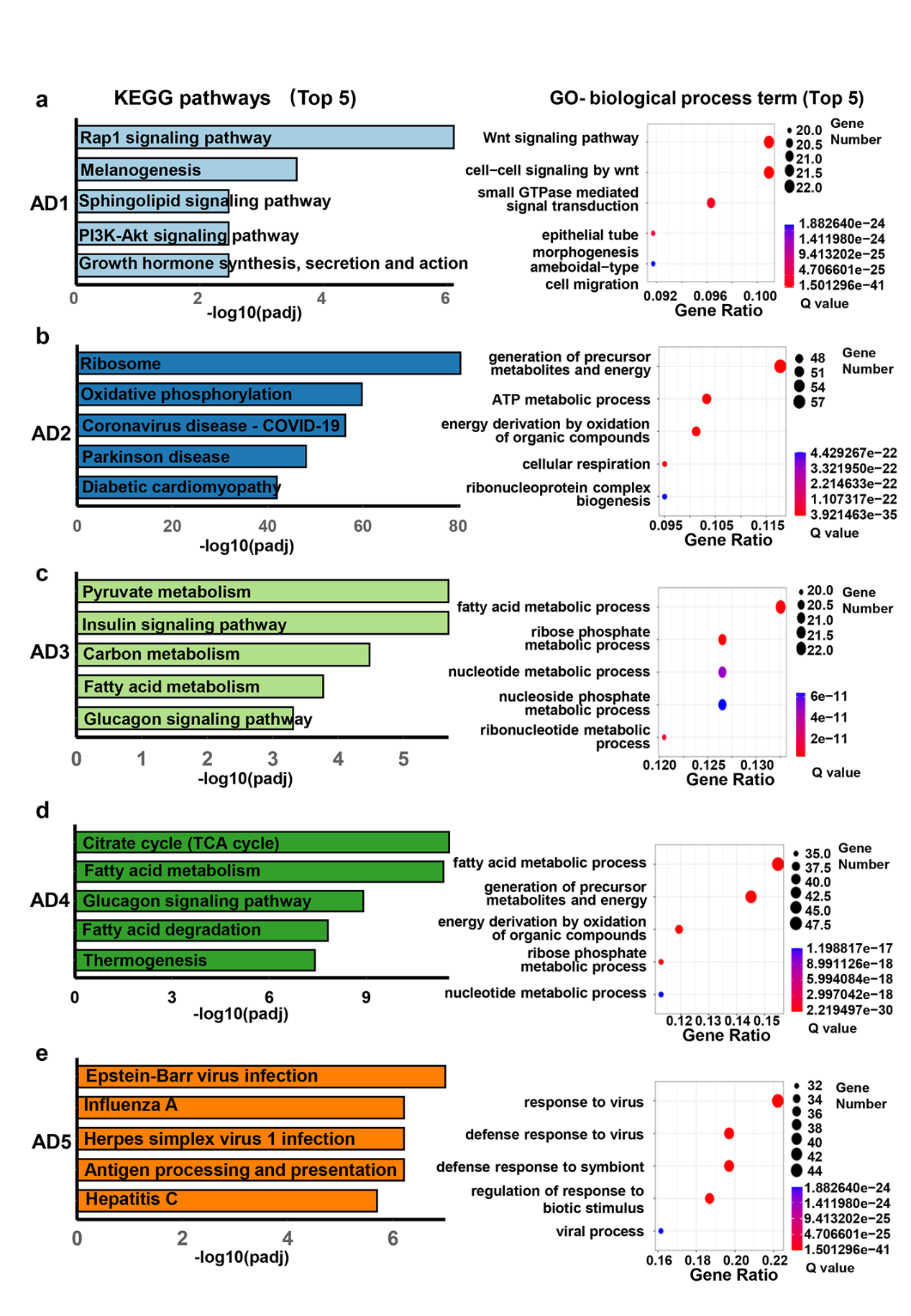


**Supplementary Figure S5** KEGG pathway analysis and biological process term of GO enrichment analysis of feature genes in AD1−AD5 adipocyte subsets. GO and KEGG pathway enrichment analysis of AD1 (a), AD2 (b), AD3 (c), AD4 (d), AD5 (e) respectively. The top 5 enriched terms are shown for the comparison.


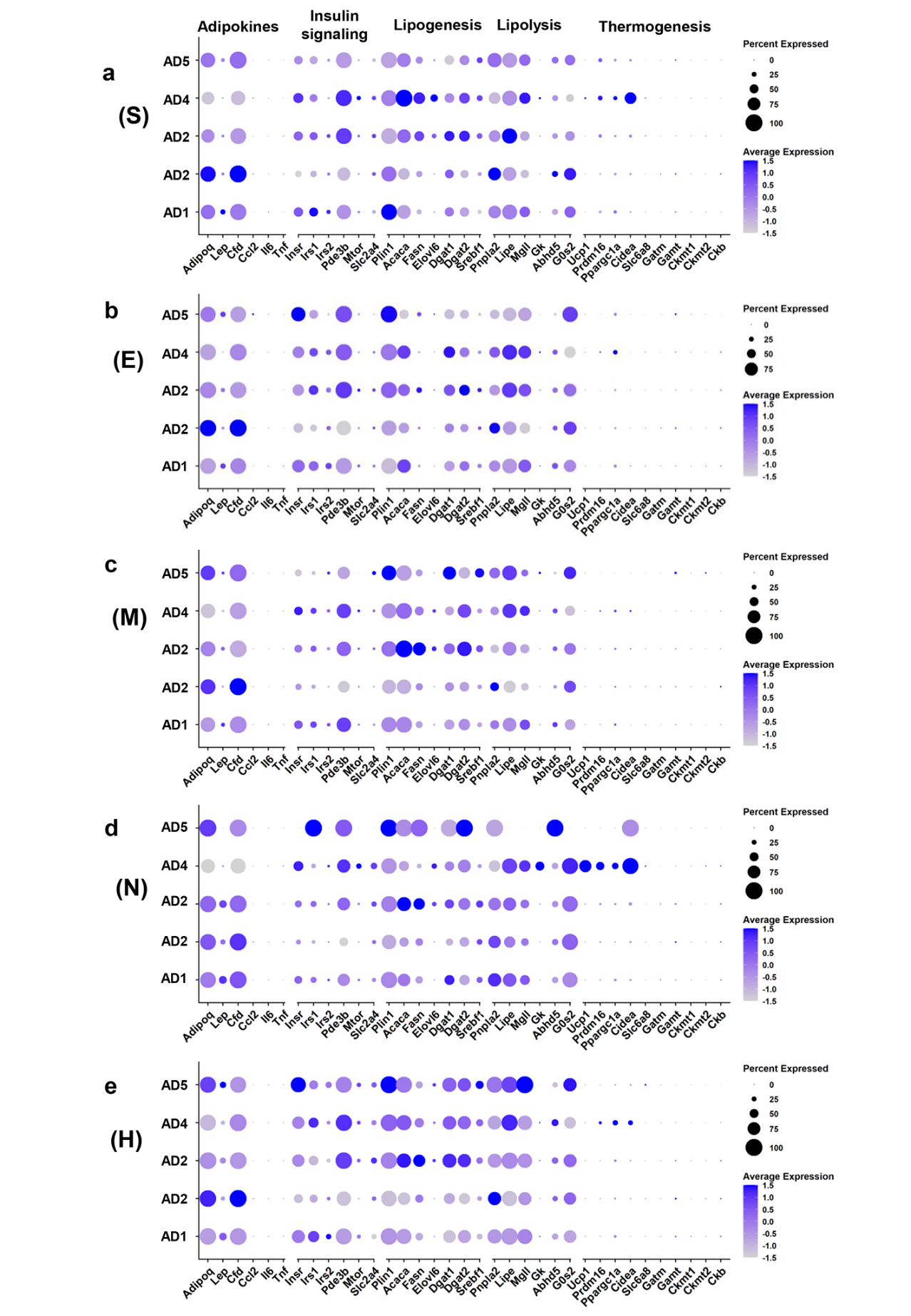


**Supplementary Figure S6** Gene expression levels related to adipokine secretion, insulin signaling, lipogenesis, lipolysis, and thermogenesis of adipocyte sub-clusters in different depots. (a) subcutaneous (S), (b) epididymal (E), (c) mesenteric (M), (d) peri-nephritic (N), and (e) peri-heart (H) adipose depots.

**Supplementary Table S1** Primer sequences for RT-qPCR. F, Forward; R, Reverse.

| **genes** | **Primer sequence** |
| --- | --- |
| Myog-F | TTCGAACCACCAGGCTACGA |
| Myog-R | TCAGCCGTGAGCAGATGATC |
| Myod-F | AGCACTACAGTGGCGACTCA |
| Myod-R | GCTCCACTATGCTGGACAGG |
| Cebpa-F | CAAGAACAGCAACGAGTACCG |
| Cebpa-R | GTCACTGGTCAACTCCAGCAC |
| Pparg-F | TCGCTGATGCACTGCCTATG |
| Pparg-R | GAGAGGTCCACAGAGCTGATT |
| Adipoq-F | TGTTCCTCTTAATCCTGCCCA |
| Adipoq-R | CCAACCTGCACAAGTTCCCTT |
| Fabp4-F | AAGGTGAAGAGCATCATAACCCT |
| Fabp4-R | TCACGCCTTTCATAACACATTCC |
